# Rethinking treatment failures. Research on a group of Italian psychotherapists

**DOI:** 10.3389/fpsyg.2024.1403736

**Published:** 2024-08-05

**Authors:** Osmano Oasi, Francesca De Salve, Chiara Rossi, Simone Maggio, Ilaria Casabona, Sara Molgora

**Affiliations:** Department of Psychology, Catholic University of the Sacred Heart, Milan, Italy

**Keywords:** psychotherapeutic failures, countertransference, emotional response, dropout, gender, treatment interruptions

## Abstract

**Introduction:**

Psychotherapeutic failures involve situational, relational, and personal factors. Dropout refers to a patient’s unilateral termination of treatment without the therapist’s knowledge or approval. Premature termination occurs when therapy is discontinued before achieving a sufficient reduction in initial problems.

**Objective:**

This study explores the role of therapist’s emotional response (countertransference), gender, psychotherapeutic orientation, and patient diagnosis in the context of psychotherapeutic failures.

**Method:**

A mixed-method approach was used. Fifty-nine Italian psychotherapists, practicing mostly privately with at least 5 years of experience, were recruited through Italian professional internet websites. The Therapist Response Questionnaire and the Impasse Interview were administered to each psychotherapist. Each therapist was asked to reflect on their last dropout patient. Quantitative (MANOVA) and qualitative analyses (textual content analysis) were conducted with SPSS and T-LAB, respectively.

**Results:**

The quantitative analyses revealed that the most frequent countertransference response was Helpless/Inadequate, with female therapists experiencing this more frequently than male therapists. The qualitative analyses identified two main factors explaining most of the variance in countertransference responses: Parental/Protective versus Hostile/Angry, and Positive/Satisfying versus Helpless/Inadequate, with Helpless/Inadequate central. Additionally, the qualitative analysis of treatment interruption methods revealed two factors explaining over 50% of the variance. Lack of communication was linked to negative themes, while mediated and direct communication were associated with positive terms. Direct communication was characterized as useful, while mediated communication was linked to dropout and attachment figures.

**Conclusion:**

Under pressure, psychotherapists’ anxiety levels increase, often managed ambivalently or avoidantly. These results suggest that awareness of psychotherapist emotional responses is important to limit psychotherapeutic failures. These findings offer valuable insights for clinical practice.

## 1 Introduction

Treatment failures remain a prevalent issue in clinical practice, affecting various intervention models and patients with diverse diagnostic characteristics or therapist-patient dynamics (Scott and Sembi, 2006), with dropout rates in psychotherapy hovering around 20% (Swift and Greenberg, 2012). The concept refers to a situation in which the therapeutic intervention does not achieve the intended goals or outcomes, resulting in the persistence or worsening of the patient’s psychological symptoms and distress (Goldberg and Stricker, 2011). Treatment failure is a complex and multifaceted phenomenon arising from a combination of factors, including inadequate assessment, mismatch between the therapeutic approach and the patient’s needs, external life stressors, coexisting medical or psychiatric conditions (De Salve et al., 2023; Rossi et al., 2023), or difficulties within the therapeutic relationship (Wierzbicki and Pekarik, 1993). Premature termination or dropout from therapy, a subset of treatment failures, is particularly concerning as it can lead to exacerbated symptoms, increased distress, and diminished hope regarding the efficacy of future therapeutic interventions (Barrett et al., 2008; von der Lippe et al., 2008; Swift and Greenberg, 2012).

From the therapist’s perspective, premature termination can be disheartening, impacting professional self-efficacy and satisfaction, and leading to feelings of failure, frustration, and helplessness (Lambert, 2013). It disrupts the therapeutic process and hinders opportunities for professional growth and learning (Norcross and Wampold, 2018). The wider community is also affected, as untreated or partially treated psychological conditions increase healthcare costs and resource allocation issues (Lutz et al., 2021). Untreated psychological issues can further lead to broader societal impacts, including decreased productivity, increased absenteeism, and greater reliance on social support systems (de Haan et al., 2013).

Numerous studies have investigated potential predictors of premature termination, including age, level of functioning, and socio-demographic characteristics (O’Keeffe et al., 2019). Significant predictors include therapist-related factors such as experience and skills (Hill and Norcross, 2023), and patient-related factors such as diagnosis (Cavalera et al., 2021), and setting-related factors (Gabbard, 2016). Notably, negative countertransference (e.g., negative perceptions of the patients, repetition of negative parental responses, unresolved negative emotions) appears to correlate with patient dropout (Tishby and Wiseman, 2022). Patient-related factors, such as borderline personality disorder, childhood trauma, and the quality of the therapeutic alliance (Steuwe et al., 2023), along with lower socioeconomic status (Wampers et al., 2018), significantly predict dropout rates. Additionally, personality traits such as perceptual dysregulation, unusual belief suspiciousness, rigid perfectionism (Berghuis et al., 2021), and emotional dysregulation (Doyle et al., 2023) are strongly associated with increased likelihood of discontinuation. Impairments in self-functioning, as outlined in Criterion A of the DSM-5 TR, also predict dropout (Busmann et al., 2019).

A systematic review by Werbart et al. (2019) emphasized the importance of therapists’ experience, training, and skills, along with their ability to provide concrete and emotional support, in reducing dropout rates. Relationship and process factors, such as the quality of the therapeutic alliance, client dissatisfaction, and pre-therapy preparation, play crucial roles. Client-initiated discontinuation is more common in dyads characterized by low early therapeutic alliance, less agreement on treatment goals and procedures, greater client dissatisfaction, and therapists with less experience and training. Additionally, low session frequency, few appointments, and frame ruptures are linked to higher dropout rates (Werbart et al., 2019). Conversely, providing concrete support and adequately preparing clients for therapy can mitigate dropout. These findings underscore the importance of maintaining stable and reliable therapeutic boundaries while flexibly adapting to meet clients’ needs to enhance treatment adherence and outcomes. However, the lack of consensus in studies examining the concept of abandonment underscores the need for further investigation (Swift et al., 2018).

Therefore, the goal of this study is to explore the characteristics of treatment failures from the perspectives of Italian psychotherapists. Using a mixed-method approach, the study collects data on therapists’ views. Quantitatively, it aims to evaluate whether therapists’ emotional responses (countertransference) differ based on therapist and patient gender, patient diagnosis, and method of treatment interruption. Qualitatively, the study seeks to obtain detailed information about countertransference and the mode by which the termination of therapy was communicated (none, in person, mediated).

## 2 Materials and methods

### 2.1 Participants

Fifty-nine Italian psychotherapists were recruited for a study through various means. These included informal contacts, a Google search engine, two professional websites that provided profiles of several psychotherapists along with their main information and curriculum vitae (accessible through URLs https://www.psicologi-italia.it and https://www.guidapsicologi.it/), and a Facebook group of psychologists and psychotherapists. All participating psychotherapists had been registered in the professional Register of Specialization in Psychotherapy for a minimum of 5 years. Among them, 25 (42.4%) were oriented toward psychodynamic therapy, while 34 (57.6%) practiced cognitive-behavioral therapy. The sample comprised 13 males (22%) and 46 females (78%), with a mean age of 44.1 years (SD = 8.5, range = 34–76).

### 2.2 Procedure

During 2018–2019, all psychotherapists were contacted either by telephone or email and invited to participate in this study. After this, a face-to-face meeting with available psychotherapists was scheduled to discuss the research project in detail. For those who agreed to participate, informed consent and consent to record the interview were obtained. The study complies with the Guidelines of the 1964 Declaration of Helsinki and its later amendment (World Medical Association, 2013), and all the study participants gave their informed consent after being properly informed.

To each psychotherapist, the Therapist Response Questionnaire (Tanzilli et al., 2016) and the Impasse Interview (Hill et al., 1996) were administered. The questionnaire and the interview were completed by therapists thinking of one specific patient who had recently interrupted psychotherapy.

Out of the total number of patients, 36 were female (61%) and 23 were male (39%). Based on DSM-IV TR diagnoses, 34 patients (57.6%) had symptomatology attributed to Axis I (anxiety, depression, PTSD, bipolar disorder), while 25 patients (42.4%) had an Axis II disorder (borderline, narcissistic, paranoid, dependent personality disorders). Nine patients (15.8%) presented comorbidities between axes. Most patients were seen in a private setting. The average number of sessions before dropout was 12.2 (median = 6; SD = 19.1; range = 1–100). Regarding the clinical process variables, in 25 (42.27%) instances the patient communicated the interruption in person, while in 17 cases (28.81%) did it through a phone call. In the last 16 cases (27.12%) no communication of the interruption by the patient was made. Additionally, 31 therapists did not conduct supervision after the interruption, while 28 did.

### 2.3 Materials

#### 2.3.1 Therapist Response Questionnaire (TRQ)

The TRQ (Tanzilli et al., 2016) is a questionnaire designed to help therapists explore their countertransference patterns during psychotherapy. The questionnaire comprises 79 items that assess various thoughts, feelings, and behaviors of the therapist toward the patient. Each item is rated on a Likert scale ranging from 1 (“not true”) to 5 (“very true”). The TRQ measures nine factors that include Helpless/Inadequate (*ω* = 0.87), Overwhelmed/Disorganized (*ω* = 0.86), Positive/Satisfying (*ω* = 0.78), Parental/Protective (*ω* = 0.71), Special/Overinvolved (*ω* = 0.70), Criticized/Devalued, (*ω* = 0.79), Hostile/Angry (*ω* = 0.85), Sexualized (*ω* = 0.85), and Disengaged (*ω* = 0.83). A higher score on any of the nine factors indicates a greater presence of the specific countertransference style measured by that factor.

#### 2.3.2 Impasse Interview

The interview (Hill et al., 1996) is designed to investigate a recent or significant clinical situation that was not resolved and led to the discontinuation of therapy. It consists of four sections that cover the following topics: (1) General information about the therapist, including their training and psychotherapeutic orientation; (2) General information regarding impasse situations experienced by the therapist; (3) General information about the patient involved in the impasse situation; (4) An in-depth analysis of the impasse with this patient. The interview is based on the definition of impasse as a difficult or stalemate situation that makes therapy progress challenging and can cause it to be interrupted. Such situations can lead to negative emotions such as anger, disappointment, and a sense of failure for both the therapist and the patient.

### 2.4 Data analyses

The data of the TRQ were analyzed using SPSS, version 29.0.1. Descriptive statistics were required to test the distribution of the variables. The normality of the distribution of the sample was assumed by observing skewness and kurtosis values that resulted within the acceptable range between −2 and +2 (Podsakoff et al., 2003). Subsequently, MANOVA was conducted to evaluate differences (Helpless/Inadequate, Overwhelmed/Disorganized, Positive/Satisfying, Parental/Protective, Special/Overinvolved, Criticized/Devalued, Hostile/Angry, Sexualized, and Disengaged) among the nine countertransference dimensions related to therapist’s gender and psychotherapeutic orientation, as well as patient’s gender and diagnosis. Bonferroni correction was applied (*p* < 0.005).

The Impasse Interview was audio-recorded and then transcribed electronically using an online transcription software and its application^1^. The *verbatim* of the interviews was analyzed with the software T-LAB (version 7.3.0). This software is based on a mixed method (i.e., quantitative, and qualitative) and permits an in-depth exploration of the texts through a wide range of algorithms and operations at both a descriptive and interpretative level. Specifically, it consists of a set of linguistic, statistical, and graphical tools useful to evaluate the relations among words (i.e., lexical units) within an entire text (i.e., the *corpus*), or within specific sections of the text (i.e., the elementary context – that is, the segmentation of the corpus automatically done by the software, or the context units – that is, the segmentation of the corpus done by the researcher based on some independent variables). T-LAB belongs to the word-driven software family and makes it possible to create new interpretable data through occurrence and co-occurrence matrices, starting from the original text. Since this software combines linguistic and statistical tools, it offers many advantages in terms of rigor and reliability of the analyses (Lancia, 2004).

For this research, the whole *corpus* was composed of 59 interviews. According to our research questions, only section 3 (general information about the identified patient) and section 4 (in-depth analysis of the impasse) were used. The analyses were performed considering all the lemmas with a frequency cut-off value of 10 or more for a total of 6,664 analyzed lemmas. Specifically, the *correspondence analysis* was performed. The correspondence analysis is a method of comparative analysis that allows for comparison between different segments of a corpus. Specifically, it permits the identification of similarities and differences among different parts of the corpus (the context units). Like factor analysis, with correspondence analysis, a set of new variables (i.e., factors) are extracted by organizing the different parts of the corpus through some spatial vectors (horizontal and vertical): the elements that are placed on opposite ends of each factor (or vector) are most different from each other. This analysis is based on the value test statistic with a threshold value for significance of ±1.96. Two different correspondence analyses were implemented. The first one was on the “Countertransference response” variable, which had four levels: helpless/inadequate, parental/protective, positive/satisfying, and hostile/angry – which are the most common types of countertransference experienced in our sample. The second correspondence analysis was on the “method of treatment interruption” variable, which had three levels: de visu (communicated during a session), mediated (through a telephone communication or with a mobile phone message), and none (failure to communicate the end of therapy by the patient).

## 3 Results

### 3.1 Quantitative results

#### 3.1.1 Therapist response questionnaire

In Table 1 the frequencies and mean values are reported for all the nine countertransference dimensions. As shown, overall, the most frequent countertransference dimension was Helpless/Inadequate. Whereas no one of the therapists involved in the research declared to feel overwhelmed/disorganized, special/overinvolved, sexualized, or disengaged emotional responses.

**Table 1:** Mean values of the 9 countertransference of the TRQ.

Types of countertransference	Frequency (percentage)	Overall Mean (SD)
Helpless/Inadequate	23 (39.0%)	3.02 (0.91)
Overwhelmed/Disorganized	0	2.34 (0.83)
Positive/Satisfying	8 (13.6%)	2.48 (0.64)
Hostile/Angry	8 (13.6%)	2.23 (0.88)
Criticized/Devalued	1 (1.7%)	2.15 (0.78)
Special/Overinvolved	0	1.58 (0.51)
Parental/Protective	19 (32.1%)	2.63 (0.87)
Sexualized	0	1.23 (0.48)
Disengaged	0	1.90 (0.65)

*N* = 59.

Tables 2, 3 show values for all nine countertransference dimensions, considering the therapist’s gender and psychotherapeutic orientation, as well as the patient’s gender and diagnosis.

**Table 2:** Mean values of the 9 countertransference of the TRQ assessed for the therapist’s gender and psychotherapeutic orientation.

Types of countertransference	Sex		Psychotherapeutic orientation	
	Male *M* (SD)	Female *M* (SD)	Psychodynamic *M* (SD)	Others *M* (SD)
Helpless/Inadequate	2.37 (0.66)	3.20 (0.90)	3.20 (0.87)	2.89 (0.94)
Overwhelmed/Disorganized	2.10 (0.68)	2.41 (0.86)	2.41 (0.72)	2.29 (0.90)
Positive/Satisfying	2.68 (0.42)	2.42 (0.68)	2.45 (0.59)	2.50 (0.67)
Hostile/Angry	2.38 (0.93)	2.19 (0.87)	2.36 (0.91)	2.15 (0.85)
Criticized/Devalued	1.81 (0.56)	2.24 (0.81)	2.35 (0.85)	2.00 (0.70)
Special/Overinvolved	1.78 (0.62)	1.52 (0.47)	1.45 (0.43)	1.67 (0.56)
Parental/Protective	2.70 (0.84)	2.61 (0.89)	2.63 (0.88)	2.63 (0.88)
Sexualized	1.25 (0.46)	1.23 (0.50)	1.32 (0.59)	1.17 (0.38)
Disengaged	1.92 (0.61)	1.89 (0.67)	1.98 (0.71)	1.83 (0.61)

*N* = 59.

**Table 3:** Mean values of the 9 countertransference of the TRQ assessed for the patients’ gender and diagnosis.

Types of countertransference	Sex		Diagnosis^1^	
	Male *M* (SD)	Female *M* (SD)	Axis 1 *M* (SD)	Axis 2 *M* (SD)
Helpless/Inadequate	2.91 (0.98)	3.09 (0.88)	2.89 (0.84)	3.21 (1.01)
Overwhelmed/Disorganized	2.12 (0.74)	2.48 (0.86)	2.21 (0.79)	2.52 (0.88)
Positive/Satisfying	2.43 (0.57)	2.51 (0.68)	2.49 (0.55)	2.45 (0.75)
Hostile/Angry	2.03 (0.82)	2.36 (0.90)	2.12 (0.92)	2.40 (0.83)
Criticized/Devalued	1.95 (0.72)	2.27 (0.80)	2.08 (0.77)	2.26 (0.82)
Special/Overinvolved	1.40 (0.39)	1.68 (0.56)	1.64 (0.58)	1.50 (0.41)
Parental/Protective	2.63 (0.91)	2.63 (0.86)	2.54 (0.85)	2.70 (0.90)
Sexualized	1.38 (0.68)	1.14 (0.27)	1.27 (0.52)	1.11 (0.30)
Disengaged	1.74 (0.62)	2.00 (0.66)	1.84 (0.63)	1.93 (0.70)

^1^*N* = 59. Diagnoses were made through DSM-IV TR.

Finally, to investigate differences in countertransference due to therapists’ gender, MANOVA was required. No main effect between the two groups was obtained (Wilks’s Λ = 0.738; *F* = 1.854; *η*_partial_ = 0.262, *p* > 0.05). All the dependent variables appeared no significant to the Levene test.

According to Bonferroni’s multiple comparison tests, significant differences were obtained between male and female in helpless/inadequate type of countertransference [*F*(1,55) = 9.141; *p* = 0.004; *η*_partial_ = 0.143]. Specifically, the helpless/inadequate type of countertransference was found to be higher in the group female therapists (*M* = 3.09) compared to male (*M* = 2.91) for *p* = 0.004. Whereas no significant difference emerged for patient’s gender (*p* = 0.184), diagnosis (*p* = 0.323), and therapist’s psychotherapeutic orientation (*p* = 0.376).

### 3.2 Qualitative results: impasse interview

**Figure 1:**
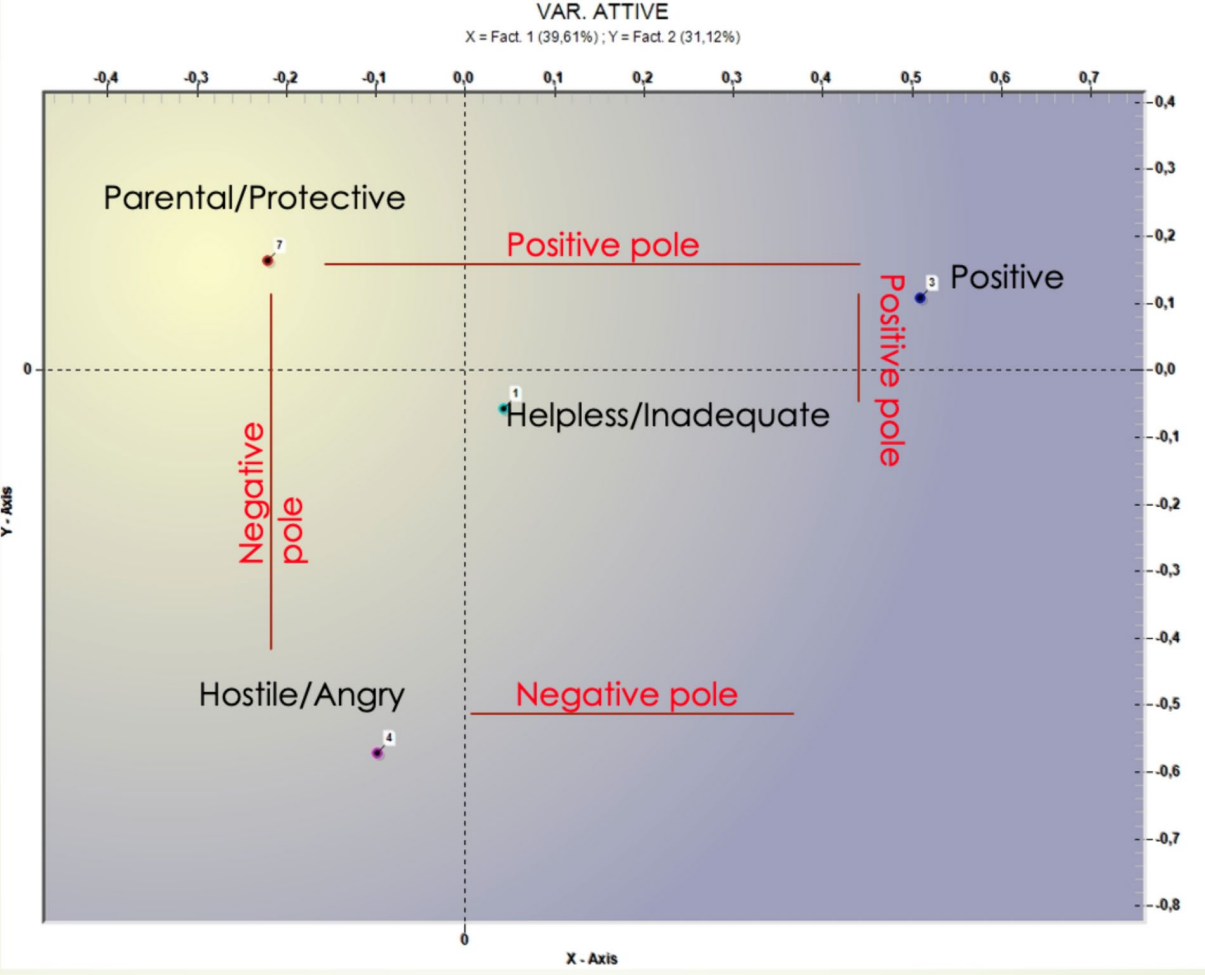
Graphical representation of the four main therapist’s emotional responses.

Two different correspondence analyses were implemented. The first was conducted on the most frequent “therapist’s countertransference response” (Helpless/Inadequate, Positive/Satisfying, Hostile/Angry, and Parental/Protective; see Table 1). This analysis showed a three-factor solution that explains, respectively, 39.6, 31.1, and 29.3% of the total variance (Table 4); but just the first two factors will be considered because they explain the largest portion of variance. From Figure 1 emerged that both factors have a positive and negative pole. On the first factor “Parental/Protective” (positive pole) is opposite to “Hostile/Angry” (negative pole) and on the second factor “Positive/Satisfying” (positive pole) is opposite to “helpless/inadequate” (negative pole). Graphically, the emotional response of “helplessness/inadequate” is placed at the “core” of the other emotional reactions. Notably, it is conceivable, that the helpless or inadequate reaction catalyzes activating attachment patterns in psychotherapists experiencing pressure (Wallin, 2013). Following the spectrum of polarities, Factor 1 emerges as a result of parental/protective and hostile/angry emotional responses. The results introduce elements of ambivalence within parental/protective emotional responses. Conversely, Factor 2 arises from a combination of parental/protective and positive emotional responses, demonstrating a tendency toward avoidance.

**Table 4:** Lemmas and test value of therapist’s emotional responses.

Factor 1				Factor 2			
Negative pole		Positive pole		Negative pole		Positive pole	
Lemmas	Test value	Lemmas	Test value	Lemmas	Test value	Lemmas	Test value
PARENTAL	−28.49	POSITIVE	36.11	HOSTILE	−32.28	PARENTAL	21.24
HOSTILE	−5.54	Shoe	5.51	Mother	−7.88	POSITIVE	7.65
Group	−5.11	Partner	5.49	HELPLESS	−6.58	Group	3.72
She	−4.98	judgment	4.81	Big	−5.88	Need	3.56
Slightly	−4.32	Crisis	4.33	Remembrance	−5.34	Difficulty	2.86
Therapy	−4.13	To return	4.16	I	−4.98	Father-in-law	2.78
Appointment	−3.81	Point	4.15	Interview	−4.88	Know	2.68
Difficulty	−3.52	Positive	4.14	Exit	−4.25	Impasse	2.51
Psychiatrist	−3.48	Uncle	4.11	Good	−4.24	Involvement	2.49
Child	−3.44	Stay	4.04	Dropout	−3.83	Clear	2.45

Qualitative analyses were conducted on 59 interviews.

**Figure 2:**
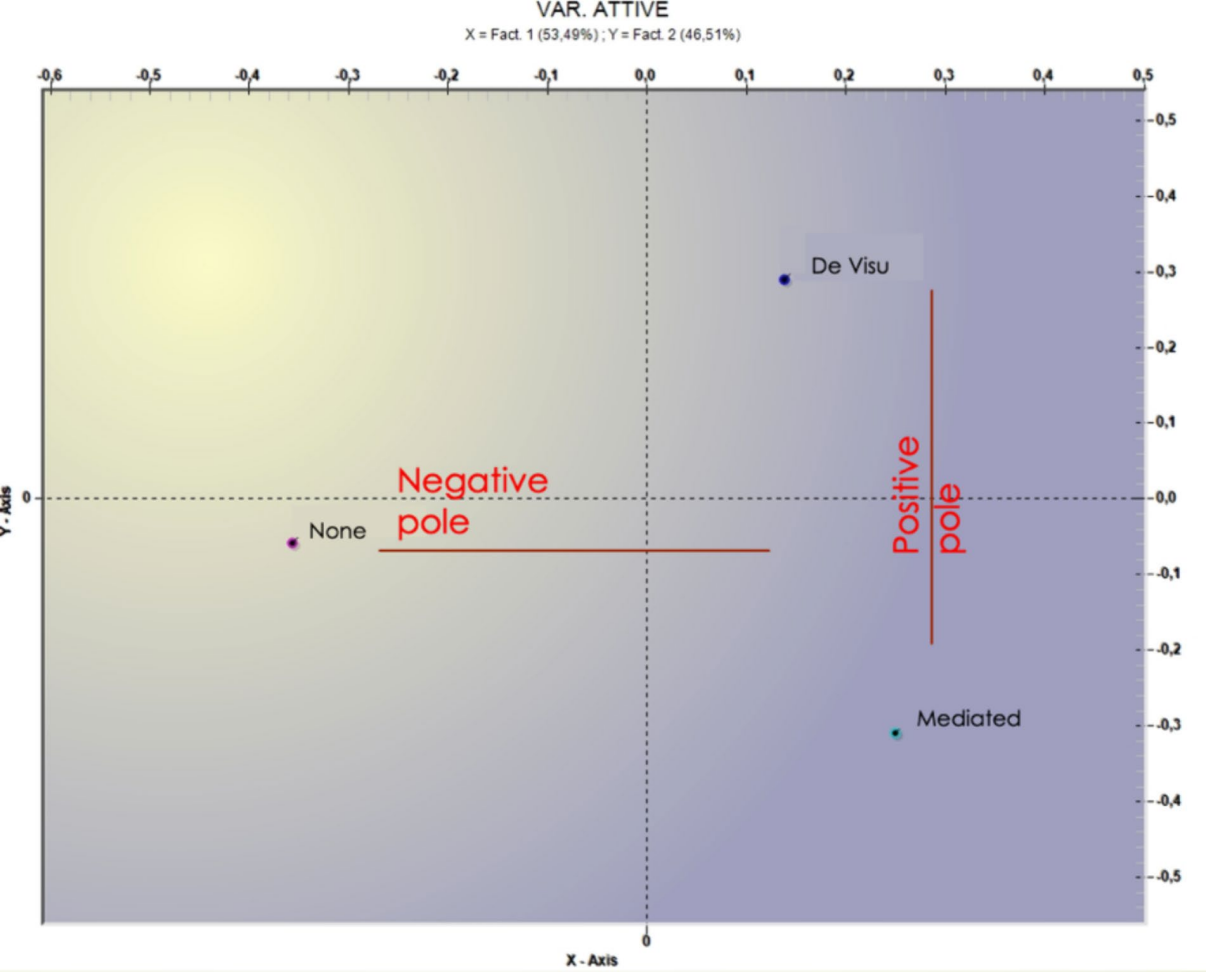
Graphical representation of the method of treatment interruption.

The analysis performed on the “method of treatment interruption” variable showed two factors explaining, respectively, 53.49 and 46.51% of the data variance (Table 5) and (Figure 2).

**Table 5:** Lemmas and test value of therapist’s emotional responses.

Factor 1				Factor 2			
Negative pole		Positive pole		Negative pole		Positive pole	
Lemmas	Test value	Lemmas	Test value	Lemmas	Test value	Lemmas	Test value
NONE	−39.74	MEDIATED	24.29	MEDIATED	−30.04	DE VISU	34.40
Appointment	−5.96	DE VISU	16.35	Speech	−7.32	I	5.61
Father-in-law	−5.10	She	6.20	Husband	−5.13	Clearly	4.31
Silence	−4.78	Mother	4.67	Mother	−4.93	Method	4.11
To disappear	−4.70	Speech	4.36	To see	−3.90	To be useful	3.97
To come	−4.47	Boyfriend	3.52	Return	−3.89	To work	3.88
Stuff	−4.41	Fear	3.52	Dropout	−3.89	Appearence	3.70
Respect	−4.21	A little	3.48	To scare	−3.81	Meeting	3.49
Money	−4.09	Psychiatrist	3.46	Own	−3.55	Analysis	3.39
Do not Know	−3.01	Good	3.25	Borderline	−3.39	Shoe	3.30

Qualitative analyses were conducted on 59 interviews.

On the first factor, “none” is opposed to “mediated” and “de visu” (Figure 1). Within the lack of any kind of communication the theme of “respect” seems to be crucial and associated with some negative action such as “to disappear,” to interrupt the psychotherapy in “silence,” whereas the presence of communication (direct or indirect) is associated with “mother” “speech,” “psychiatrist, and “good.” On the second factor, instead, it is possible to capture the differences between direct (i.e., *de visu*) and indirect (i.e., mediated) interruption communication. Direct communication seems to be characterized “useful” and “meeting,” whereas mediated communication is associated with lemmas that refer to “dropout” or attachment figure (i.e., husband and mother). In Table 5 the most significant terms and the respective test values are reported.

## 4 Discussion

The present study had two main aims. Quantitatively, the objective was to evaluate whether therapists’ emotional response (countertransference) differences due to therapist and patient gender, patient diagnosis, and method of treatment interruption. Qualitatively, the goal was to obtain more detailed information about the countertransference and the mode by which the termination of therapy was communicated (none, *de visu*, mediated).

The descriptive statistics analysis revealed that therapists participating in the study commonly encounter various types of countertransference when dealing with patients who discontinue treatment. The most frequent were Helpless/Inadequate (39%) and Parental/Protective (32.1%).

Concerning the latter, Gelso and Hayes (2007) explored that the phenomenon occurs when therapists project their own parental instincts onto their patients, often in response to perceived vulnerability or neediness. Considering this definition, the results can be explained through McWilliams’s (2011, 2021) theories, which discuss how such feelings can be particularly intense when clients evoke characteristics reminiscent of the therapist’s own children or unresolved parenting issues. Pearlman and Saakvitne (1995) further elaborate on the challenges therapists face when clients’ trauma and dependency trigger their protective instincts. Racker (1968) provides a foundational understanding of these dynamics, highlighting the importance of therapists recognizing and managing their emotional responses to maintain professional boundaries. Winnicott’s (1965) concept of the therapist as a “holding environment” underscores the delicate balance therapists must achieve between offering support and avoiding over-involvement driven by parental protective impulses. This countertransference, while common, necessitates careful reflection and supervision to ensure effective therapeutic outcomes.

Based on the descriptive statistics, a MANOVA was implemented to test whether the therapist’s gender produced a difference in the type of countertransference most frequently experienced. The MANOVA results indicate a noteworthy inclination among female therapists to experience the emotional response of Helpless/Inadequate more frequently compared to their male counterparts. The Helpless/Inadequate reaction is characterized by feelings of inadequacy, incompetence, hopelessness, and anxiety, as outlined by Tanzilli et al. (2016). Therapists often experience helpless and inadequate countertransference when working with clients whose issues seem overwhelming or resistant to intervention. This reaction is particularly common with clients who present with severe trauma, chronic mental health conditions, or persistent self-destructive behaviors (Pearlman and Saakvitne, 1995). Such feelings of inadequacy and helplessness may arise from the therapist’s own unresolved issues or from a lack of effective therapeutic tools and strategies to address the client’s complex needs (Gelso and Hayes, 2007). Consequently, this form of countertransference can impede the therapeutic process, highlighting the necessity for therapists to seek supervision and engage in continuous professional development to enhance their coping mechanisms and therapeutic skills (McWilliams, 2011).

The gender disparity is undoubtedly a multifaceted issue (Tanzilli et al., 2022). In this regard, Staczan et al. (2017) identified gender-related variances in therapeutic approaches, revealing that male therapists exhibited a proclivity for employing confrontative interventions, characterized by interpretations of defense mechanisms and resistance. Conversely, female therapists demonstrated a tendency to engage in interventions characterized by empathy and support. According to recent studies, one plausible interpretation of the gender difference in countertransference suggests that a greater emphasis on confrontative interventions elicits more pronounced reactions in the therapist, and presumably, also in the patient, compared to situations where the therapist adopts a more empathic stance (Berg et al., 2019; Chertoff et al., 2020). It is conceivable that the inclination to utilize therapeutic approaches centered on empathetic validation could generate, among woman therapists, sentiments of inadequacy and helplessness toward dropout.

The results obtained by qualitative analysis may shed light on this aspect. The software organized the verbatim concerning the therapist’s countertransference response into two different factors. Factor 1 (Negative Pole) could suggest that the psychotherapist has mixed feelings, while factor 2 (Positive Pole) could indicate their tendency to avoid certain emotions. A hypothesis is that when psychotherapists are under pressure due to psychotherapeutic failure, their anxiety levels tend to increase, and they are inclined to respond to it in an ambivalent or avoidant manner (in particular, referring to Helpless/Inadequate emotional response, whom role is dominant).

Lastly, the latest correspondence analysis, implemented using the variable “method of treatment interruption” (three levels: none, mediated, de visu) revealed two factors. Factor 1 (Negative Pole) could suggest that the patients seek distance and do not seem to be interested in a closure of the therapy involving physical presence in the session. In this case, avoidance is enacted by the patient. Factor 2 (Positive Pole) could indicate an attempt to end the therapeutic relationship, either through a phone call or live-in session. Along this line, seeking contact can be interpreted as an attempt to end a relationship maturely (Joyce and Piper, 1998).

Despite the current study’s contributions to understanding the dropout phenomenon from the therapists’ perspective, several limitations must be acknowledged. Firstly, the study solely reflects the therapists’ viewpoints, and inter-rater reliability for the diagnoses was not established. Secondly, the sample is limited and predominantly female, which may affect the generalizability of the findings. Additionally, the study lacks information on the therapists’ sexual orientation and supervision during treatment, which are uncontrolled variables that could influence the results. Finally, the research design employed does not permit causal inferences. Consequently, the findings should be interpreted with caution.

Nevertheless, to the best of our knowledge, the study presented is the first in the Italian context to propose a mixed-method approach to investigate therapists’ perspectives on psychotherapeutic failure. While mixed-method studies are suggested for investigating complex phenomena (Creswell, 1999) such as therapeutic failure, several limitations must be taken into consideration. Undoubtedly, considering the limitations, future studies should be conducted on a larger, gender-balanced sample. Most studies in the literature take the patient’s or therapist’s point of view into account. Longitudinal studies taking the patient-therapist dyad as the unit of measurement could provide comprehensive information on the process and the predictor variables of therapeutic failure.

### 4.1 Clinical implications

The study’s results have some important clinical implications. Already according to Freud’s (1912) theories, therapists may misunderstand cues related to the progress of psychotherapy, which can lead to disruptions in treatment. When working with patients who have limited insight and express a desire for specific changes, therapists face the challenge of transforming abstract ideas into realistic goals that address the fundamental issues requiring psychotherapeutic intervention. This highlights the importance of therapists being able to navigate complex situations and translate abstract desires into achievable objectives to improve treatment outcomes. Therefore, it is increasingly accepted that therapists’ ability to recognize their reactions to patients can enhance their sensitivity in diagnosing patients, lead to more accurate case formulations, and contribute to more successful therapeutic interventions (Gabbard, 2009; Bateman and Fonagy, 2016; Lingiardi and McWilliams, 2017).

In conclusion, countertransference is considered a central component of the therapeutic process capable of impacting outcome measures (Abargil and Tishby, 2022). It should be noted that countertransference management does not reduce the number of its manifestations in therapy, but it leads to better therapeutic outcomes, for this reason, it is conceivable that psychotherapist emotional response awareness is important to limit psychotherapeutic failures (Abargil and Tishby, 2022).

## 5 Conclusion

The challenge of treatment failure is a persistent problem in clinical practice and research. Supervision and attention to countertransference in psychotherapy are essential strategies for mitigating dropout rates (Barrett et al., 2008). Through supervision, therapists enhance their competence by addressing blind spots and biases, while also identifying and managing countertransference reactions. This process fosters a stronger therapeutic alliance (Sharf et al., 2010; Sijercic et al., 2021) and enables therapists to detect and address patients’ concerns early on, leading to tailored treatment planning (Cooper et al., 2023). By integrating client feedback and adjusting interventions accordingly, supervision contributes to improved treatment outcomes and reduced dropout rates in psychotherapy (Agass, 2002; Roos and Werbart, 2013).

## Data availability statement

The raw data supporting the conclusions of this article will be made available by the authors, without undue reservation.

## Ethics statement

Ethical approval was not required for the studies involving humans because the study was conducted on a sample of professional psychotherapists. Participation was voluntary after reviewing and signing the informed consent form, which explicitly highlighted the option to withdraw from the study at any time. The studies were conducted in accordance with the local legislation and institutional requirements. The participants provided their written informed consent to participate in this study.

## Author contributions

OO: Conceptualization, Project administration, Supervision, Writing – review & editing. FDS: Writing – original draft, Writing – review & editing. CR: Writing – review & editing. SMa: Data curation, Methodology, Writing – original draft. IC: Data curation, Writing – original draft. SMo: Formal analysis, Methodology, Software, Supervision, Writing – review & editing.

## Footnotes

^1^ transcribe.wreally.com
